# The miR-1290/OGN axis in ovarian cancer-associated fibroblasts modulates cancer cell proliferation and invasion

**DOI:** 10.1186/s13048-024-01364-w

**Published:** 2024-02-24

**Authors:** Biyao Jiang, Songshu Xiao, Shan Zhang, Fang Xiao

**Affiliations:** Department of Obstetrics and Gynecology, The Third Xiangya Hospital of Central South University, NO.138 Tongzipo Road, Yuelu District, Changsha, Hunan 410013 China

**Keywords:** Ovarian cancer, cancer-associated fibroblasts (CAFs), miR-1290, OGN, The Akt/mTOR pathway

## Abstract

Despite receiving first-line treatment, ovarian cancer patients continue to experience a high rate of recurrence; nearly all women with ovarian cancer develop chemoresistance and succumb to the disease. In this study, cancer-associated fibroblasts (CAFs) and normal fibroblasts (NFs) were isolated from tumor-containing and normal omenta, respectively, and the downregulation of osteoglycin (OGN) in CAFs was observed. OGN overexpression in CAFs significantly inhibited ovarian cancer cell viability, DNA synthesis, and cell invasion. OGN overexpression also changed epithelial-mesenchymal transition (EMT) markers and promoted mTOR and Akt phosphorylation in ovarian cancer cells. miR-1290 targeted OGN and inhibited OGN expression. miR-1290 overexpression in CAFs significantly promoted ovarian cancer cell viability, DNA synthesis, and cell invasion. Moreover, miR-1290 overexpression in CAFs also changed EMT markers and promoted mTOR and Akt phosphorylation within ovarian carcinoma cells. Finally, when ovarian cancer cells in a conditioned medium derived from CAFs co-transduced with miR-1290 mimics and OGN-OE were cultured, the effects of miR-1290 overexpression were partially reversed by OGN overexpression. In nude mouse xenograft tumor models, OGN overexpression in CAFs suppressed tumor growth, whereas miR-1290 overexpression in CAFs increased tumor growth. In conclusion, a miRNA/mRNA axis in ovarian cancer CAFs modulating the proliferative and invasive abilities of ovarian cancer cells, possibly via the Akt/mTOR pathway, was demonstrated.

**Supplementary Information:**

The online version contains supplementary material available at 10.1186/s13048-024-01364-w.

## Introduction

Despite the fact that improved surgical techniques and enhanced chemotherapeutic regimens have increased the survival rate of patients with advanced ovarian cancer in the last two decades, nonselective cytotoxic drug therapy frequently results in severe toxic effects and transient antitumor responses [1]. Ovarian cancer patients continue to experience a high rate of recurrence even when receiving first-line treatment; nearly all women diagnosed with the disease will develop chemoresistance and succumb to the disease [2]. Cancer-associated fibroblasts (CAFs) are the main stromal cell types in ovarian cancer tissues [3]. They undergo an activation process associated with the secretion of growth factors, cytokines, and CAF-specific proteins and induce the production of extracellular matrix (ECM) proteins that promote the growth of tumor cells and the formation of new blood vessels and confer resistance to chemotherapy [4–7]. Thus, the investigation of the molecular and genetic alterations within ovarian CAFs would lead to the development and evaluation of targeted therapies for ovarian cancer.

Osteoglycin (OGN) showed to be decreased within multiple malignancies, such as stomach cancer [8], cervical squamous carcinoma, vaginal cancer [9], colorectal adenoma [10], invasive ductal breast carcinoma [11], and laryngeal carcinoma [12]. Cell-matrix adhesion plays a vital role in the migrating and invading capacities of tumor cells [13–15]. Being a matrix molecule, OGN might play a role in the ability of tumor cells to adhere, thereby affecting their capacity to migrate and invade, as exemplified by our study. Reportedly, OGN could inhibit the capacity of tumor cells to proliferate and invade through the PI3K/Akt/mTOR signaling in breast carcinoma [16]. In colorectal cancer, OGN reversed epithelial to mesenchymal transition (EMT) and invasive ability by the EGFR/Akt signaling [17]. Although several datasets from Gene Expression Omnibus (GEO), such as GSE40595, GSE35250, and GSE14407, indicate the downregulation of OGN in ovarian cancer, the specific effect of OGN on ovarian cancer remains unclear.

MicroRNAs (miRNAs) are a class of short, single-stranded, endogenous RNAs of 19–25 nucleotides (nts) in length derived from 70 to 100 nucleotide hairpin pre-miRNA precursors [18]. miRNAs could interact with the 3′ untranslated region (3′ UTR) of target mRNAs to suppress gene expression in a sequence-specific manner post-transcriptionally [19]. By targeting multiple downstream mRNAs, miRNAs contribute to numerous processes in carcinogenesis, such as proliferative, invasive, migratory abilities, metabolism of cancer cells, and angiogenesis [20–22]. Several research groups have recently observed changes in miRNA expression in ovarian cancer and studied their roles in ovarian carcinogenesis [23, 24]. Thus, miRNAs might also be potential detection, prognostic, diagnostic, and therapeutic targets, and markers.

This study isolated and identified CAFs and normal fibroblasts (NFs). OGN expression within CAFs and NFs and the effects of conditioned culture medium derived from CAFs (CAFs or OGN-overexpressed CAFs) or NFs on ovarian cancer cells were examined. miRNAs that might target OGN were subsequently analyzed, and miR-1290 were selected. The predicted miR-1290 targeting OGN was examined. The effects of conditioned culture medium derived from CAFs (miR-1290-overexpressed or miR-1290-inhibited CAFs) on ovarian cancer cells were examined. Finally, the effects of conditioned culture medium derived from CAFs co-transduced with OGN-OE and miR-1290 mimics on ovarian cancer cells were examined. Altogether, a miR-1290/OGN axis in CAFs in ovarian cancer, which might affect ovarian cancer cell phenotypes was demonstrated.

## Materials and methods

### Clinical specimens

Biopsy specimens of OC and surrounding non-malignant tissues were harvested from 31 patients treated at our hospital. The surrounding tissues were 3 cm away from the malignancy fringe with no obvious malignant cells, as assessed by an expert pathologist. For CAF and NF isolation, tumor-containing omentum was harvested from patients with ovarian cancer and normal omentum was harvested from female benign patients. All specimens were immediately frozen at − 80 °C after surgery using liquid nitrogen. No patients had undergone radiation therapy or chemotherapy prior to surgery. All methods were performed in accordance with the relevant guidelines and regulations (Declaration of Helsinki). Full informed consent was acquired from each participant, and approval was obtained from the Research Ethics Committee of The Third Xiangya Hospital of Central South University (No. 2020-S044).

### Isolation of primary cells

CAFs were isolated from tumor-containing omental tissue of patients with ovarian cancer following the aforementioned methods [25, 26]. Normal omental fibroblasts (NFs) were isolated from omental tissue from female patients undergoing surgery for non-malignant conditions. CAFs and NFs were isolated and confirmed as previously described [25–27]. The tissues were thoroughly rinsed with PBS, minced, and digested for 12–18 h with collagenase (3 mg/ml) and hyaluronidase (0.5 mg/ml) in 10% fetal bovine serum (FBS, Invitrogen, Carlsbad, CA, USA) in DMEM (Gibco, Carlsbad, CA, USA). Primary NFs or CAFs adhered to the tissue culture plastic within 24 h. CAF- or NF-derived culture medium (CAF-CM/NF-CM) was used for the co-culture of cancer cells.

### Immunofluorescent (IF) staining

IF staining was performed by using primary antibodies against α-SMA (55135-1-AP, Proteintech, Wuhan, China) or vimentin (10366-1-AP, Proteintech) and FITC-labeled goat against rabbit IgG as the secondary antibody for the validation of isolated CAFs and NFs. The target cells were grown on coverslips plated in DMEM with FCS and incubated for 24 h to allow efficient attachment. The cells were subsequently rinsed in PBS, fixed in 4% paraformaldehyde, permeabilized in triton-100, and blocked in PBS buffer with 5% bovine serum albumin. The coverslips were sequentially incubated with the above-mentioned primary antibodies (1:100) and FITC-labeled secondary antibodies (1:400) in a blocking buffer. Finally, the cells were examined under a fluorescence microscope (Axio Vert 200, Carl Zeiss, Jena, Germany).

### Flow cytometry

For the validation of CAFs and NFs, the isolated primary NFs or CAFs labeled with fluorophore-conjugated monoclonal antibodies, including anti-vimentin (ab194719, Abcam, Cambridge, MA, USA), anti-PDGFRβ (ab275625, Abcam), anti-EpCAM (ab112068, Abcam), and anti-CD31 (ab33858, Abcam). Flow cytometry was performed using a BD Influx instrument (BD Biosciences, Franklin Lakes, NJ, USA).

### Quantitative real-time PCR (qRT-PCR)

Total RNA was extracted from target tissue samples or cells with TRIzol reagent (Invitrogen) following the protocols. RNA samples (2 µg each) were reverse-transcribed to synthesize cDNA. The mRNA expression levels of related factors were evaluated by qRT-PCR as directed by the manufacturer on an ABI 7500 Real-Time PCR System (Thermo Fisher Scientific, Waltham, MA, USA). The primer sequences used in qRT-PCR are listed in Table S1.

### Immunoblotting

Target tissue samples or cells were homogenized in ice-cold RIPA lysis buffer containing 1% protease inhibitor for 30 min. The lysates were centrifuged, and the supernatants were then recovered. The protein concentrations were measured with a bicinchoninic acid protein assay kit. The protein samples were then applied for sodium dodecyl sulfate-polyacrylamide gel electrophoresis (SDS-PAGE). The separated proteins were transferred to a polyvinylidene fluoride membrane. The membranes were firstly blocked with 5% non-fat milk and then hybridized with the following antibodies: α-SMA (55135-1-AP; Proteintech, Wuhan, China), collagen I (66761-1-Ig, Proteintech), E-cadherin (20874-1-AP, Proteintech), vimentin (10366-1-AP, Proteintech), OGN (A07061, Boster, Pleasanton, CA, USA), p-mTOR (ab109268, Abcam), mTOR (ab2732, Abcam), Akt (Y409094, ABM, New York, NY, USA), p-Akt (Y011054, ABM) at 4 °C overnight. Horseradish peroxidase-conjugated secondary antibodies and ECL were used for detection and the protein expression was normalized to that of β-actin.

### Cell lineage and cell culture

Human ovarian cancer cell line SKOV3 (HTB-77) was procured from ATCC (Manassas, VA, USA) and cultured in McCoy’s 5a Medium Modified (30-2007, ATCC) supplemented with 10% FBS. Human ovarian cancer cell line A2780 (93,112,519) was obtained from Sigma-Aldrich (St. Louis, MO, USA) and cultured in RPMI-1640 medium supplemented with 10% FBS. All the cells were cultured in 5% CO_2_ at 37 °C.

### Cell transfection

OGN overexpression was achieved in target cells by transfecting vector containing OGN fragment (OGN-OE, ORI-Bio, Changsha, China) based on pcDNA3.1. miR-1290 overexpression or inhibition was achieved by transfecting miR-1290 mimics or inhibitors (GenePharma, Shanghai, China). All transductions were performed with the help of Lipofectamine 3000 Transfection Reagent (Thermo Fisher Scientific). The sequences of overexpressing vector, miR-1290 mimics or inhibitors are listed in Table S1.

### Cell viability by MTT assay

The transfected or co-cultured cells were counted and seeded in 96-well plates. At 0 and 48 h after seeding cells, 20 µL of 5 mg/mL MTT solution (Sigma-Aldrich) was added to each well, and the cells were incubated for 3 h. After discarding the supernatant, 150 µL of DMSO was added to dissolve the formazan. A microplate reader was used to detect the absorbance at 490 nm.

### DNA synthesis by EdU

Cells transduced or co-cultured were incubated with EdU for 2 h. The cells were subsequently fixed with 4% formaldehyde, washed with PBS, and permeated. After Apollo staining and DAPI staining, the number of cells stained with EdU was counted under a fluorescence microscope, and representative images were captured.

### Cell invasion by Transwell

A total of 5 × 10^4^ cells were seeded into the upper chambers pre-coated with Matrigel (Corning, Tewksbury, MA, USA) in each chamber. Serum-free medium (200 µl) was added at the upper compartment and 600 µl of medium with 10% FBS was added to the bottom chamber. After incubation for 48 h, the cells lying above the filter were wiped off with a cotton swab, while the invaded cells on the bottom surface were fixed, stained with crystal violet, and photographed.

### Methylation‑specific PCR (MSP) assay

Cellular DNA isolation, Genomic DNA Methylation modification, and MSP were performed to determine the methylated and unmethylated alleles of the miR-1290 promoter region as previously depicted [28]. The MSP reaction was carried out in a 20 µl system (MSP kit, Tiangen, Beijing, China), and each reaction used (200 ng of template DNA. Water was used as a negative control. The PCR primers used for MSP are listed in Table S1.

### Dual-luciferase reporter assay

The binding sequences of miR-1290 on OGN were cloned into psi-Check2 vectors (Promega, Madison, WI, USA); a mutant-type vector was constructed by mutating the predicted miR-1290 binding site. 293T cells were then co-transfected with miR-1290 mimics/inhibitors and the wild- or mutant-type reporter vectors. The cells were harvested at 48 h after transfection, and the luciferase activities were measured with the Dual-Luciferase Reporter Assay System (Promega). The relative luciferase intensity was normalized to renilla luciferase activity.

### Fluorescence in situ hybridization (FISH)

The FISH assay was performed as per the instructions of the FISH kit (RiboBio, Guangzhou, China) to test miR-1290 and OGN expression in CAFs and NFs. The miR-1290 FISH probe (sequence: TCCCTGATCCAAAAATCCA) and OGN mRNA FISH probe (sequence: GTGCTGGCTTTATCAGAGGCACAAGCAGTAACA) were designed and synthesized by GenePharma. Briefly, 4% formalin was applied to fix CAFs and NFs for 15 min. After permeabilization with Triton-X100 and digestion with proteinase K, the cells got prehybridized with a hybridization buffer at 37 °C for 30 min. Next, CAFs and NFs were incubated with a FAM-labeled miR-1290 probe (green) and a Cy3-labeled OGN probe (red) in a hybridization solution at 4 °C overnight, after which DAPI (blue) was utilized to stain the nuclei. Images were captured under a fluorescence microscope.

### Tumor xenograft model of nude mice

Animal experimental procedures were carried out with the approval of the Medicine Animal Welfare Committee of Third Xiangya Hospital of Central South University. CAFs stably expressing miR-1290 and OGN by lentivirus infection were established and then selected with geneticin. 30 female BALB/c nude mice were procured from SJA Laboratory Animal CO., LTD (Changsha, China) and were divided into five groups (*n* = 6 per group): the SKOV3 cells + CAFs group, the SKOV3 cells + CAFs (lv-NC + NC mimics) group, the SKOV3 cells + CAFs (lv-OGN + NC mimics) group, the SKOV3 cells + CAFs (lv-NC + miR-1290 mimics) group, the SKOV3 cells + CAFs (lv-OGN + miR-1290 mimics) group. 3 × 10^6^ SKOV3 cells mixed with 3 × 10^6^ transfected CAFs were suspended in 200 µL Matrix-Gel-PBS solution (Beyotime), and then the cell suspension was injected into the left flanks of the nude mouse. The volume of the tumor was measured every five days from the 10th day of the experiment. After 30 days, the mice were euthanized, and the tumors were excised and weighed. The samples of tumor tissue were collected for follow-up experiments.

### Immunohistochemical (IHC) staining

Mice tumor tissues were paraffin-embedded, cut into 4-µm thick slices, and routinely dewaxed with dimethylbenzene and then rehydrated. The antigen was repaired through a water-bath of repair solution. Then, slices were incubated in H_2_O_2_ in methanol at 26 °C for 30 min to neutralize endogenous peroxidase. The nonspecific proteins were blocked by incubation with 5% goat serum at 26 °C for 40 min. Then, the slices were incubated with primary antibody against vimentin (10366-1-AP, Proteintech) and Ki-67 (28074-1-AP, Proteintech) at 4 °C for 8 ~ 10 h. After the incubation, the slices were then washed and stained with a rabbit IgG-immunohistochemical SABC kit (Boster, Wuhan, China). After washing with PBS, hematoxylin was added for nuclei staining (0.1% Mayer’s hematoxylin). The slides were observed under a microscope after completing standard procedures.

### Data processing and statistical analysis

The data of three independent experiments are expressed as means ± SD. The Student’s *t*-test or one-way analysis of variance (ANOVA) followed by Tukey’s post-hoc was used for statistical analysis. The significance level is based on the probability of *P* < 0.05 or *P* < 0.01. SPSS 17.0 statistical software was used for all the analyses.

## Results

### Isolation and identification of cancer-associated fibroblasts (CAFs) and normal fibroblasts (NFs)

**Fig. 1:**
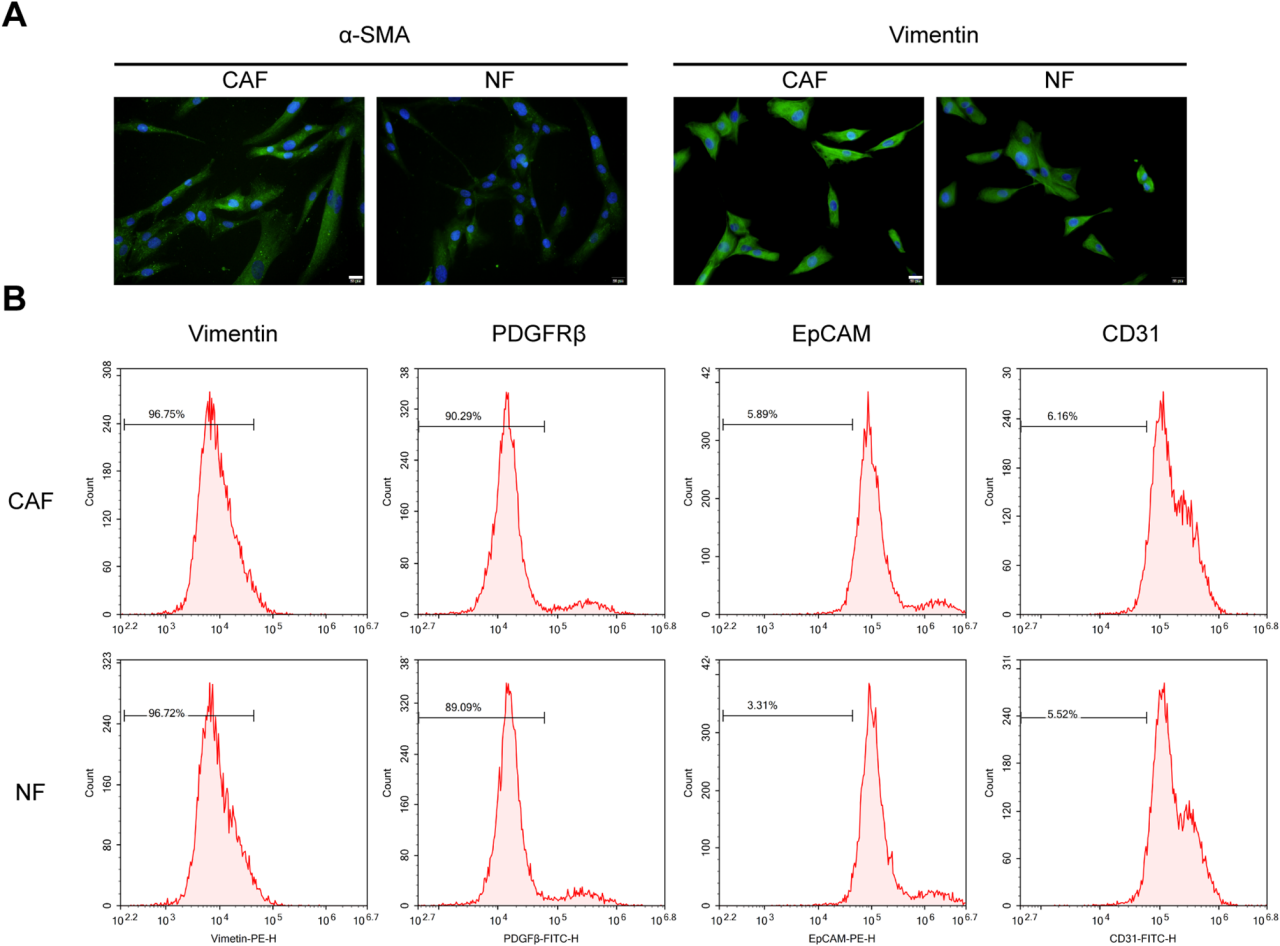
Isolation and identification of cancer-associated fibroblasts (CAFs) and normal fibroblasts (NFs) CAFs and NFs were isolated from tumor-containing omental tissue of patients with ovarian cancer and omental tissue from benign female patients, respectively, as described. Then, CAFs and NFs were identified using Immunofluorescent (IF) staining with anti-α-SMA and anti-vimentin (**A**) and Flow cytometry examining vimentin, PDGFRβ, EpCAM, and CD31 (**B**)

Firstly, CAFs and NFs were isolated from tumor-containing omental tissue of ovarian cancer patients and omental tissue from benign female patients, respectively, as described. To authenticate the isolated CAFs and NFs, IF staining was performed with anti-α-SMA and anti-vimentin, and Flow cytometry was conducted with anti-vimentin, anti-PDGFRβ, anti-EpCAM, and anti-CD31. As shown in Fig. 1A, CAFs were more abundant with α-SMA compared with NFs; as shown in Fig. 1B, NFs and CAFs were vimentin-positive, PDGFRβ-positive, EpCAM-negative, and CD31-negative.

### Culture with OGN-overexpressing CAFs-derived conditioned medium inhibits ovarian cancer cell aggressiveness

Considering the downregulation of OGN in several cancers and its anti-tumor function [16, 17, 29], online datasets were analyzed to confirm OGN expression in ovarian cancer. GSE40595 and GSE38666 (Fig. S1A-B) showed differentially expressed genes between normal and ovarian cancer stroma samples; the differentially expressed genes overlapped in 37 upregulated and 2 downregulated genes (Fig. S1C), including OGN. Differentially expressed genes were applied for GO and KEGG analysis which were enriched in cell adhesion regulation, extracellular matrix remodeling, and transmembrane transport; OGN was correlated with cell adhesion regulation, epithelial cell proliferation, differentiation, metabolism and other processes (Fig. S1D-E). According to GSE38666, OGN was shown to be dramatically downregulated within cancer stroma than that in normal stroma (Fig. S2A). According to GSE40595, OGN was significantly downregulated within ovarian cancer stromal cells compared to the normal control (Fig. S2B). According to GSE35250, OGN was significantly downregulated in normal fibroblasts co-cultured with ovarian cancer cells (Fig. S2C). According to GSE40595, OGN was shown to be dramatically downregulated within the tumor epithelium than in a normal ovarian surface epithelium (Fig. S2D). According to GSE14407, OGN was shown to be remarkably downregulated within the tumor epithelium than in a normal ovarian surface epithelium (Fig. S2E).

**Fig. 2:**
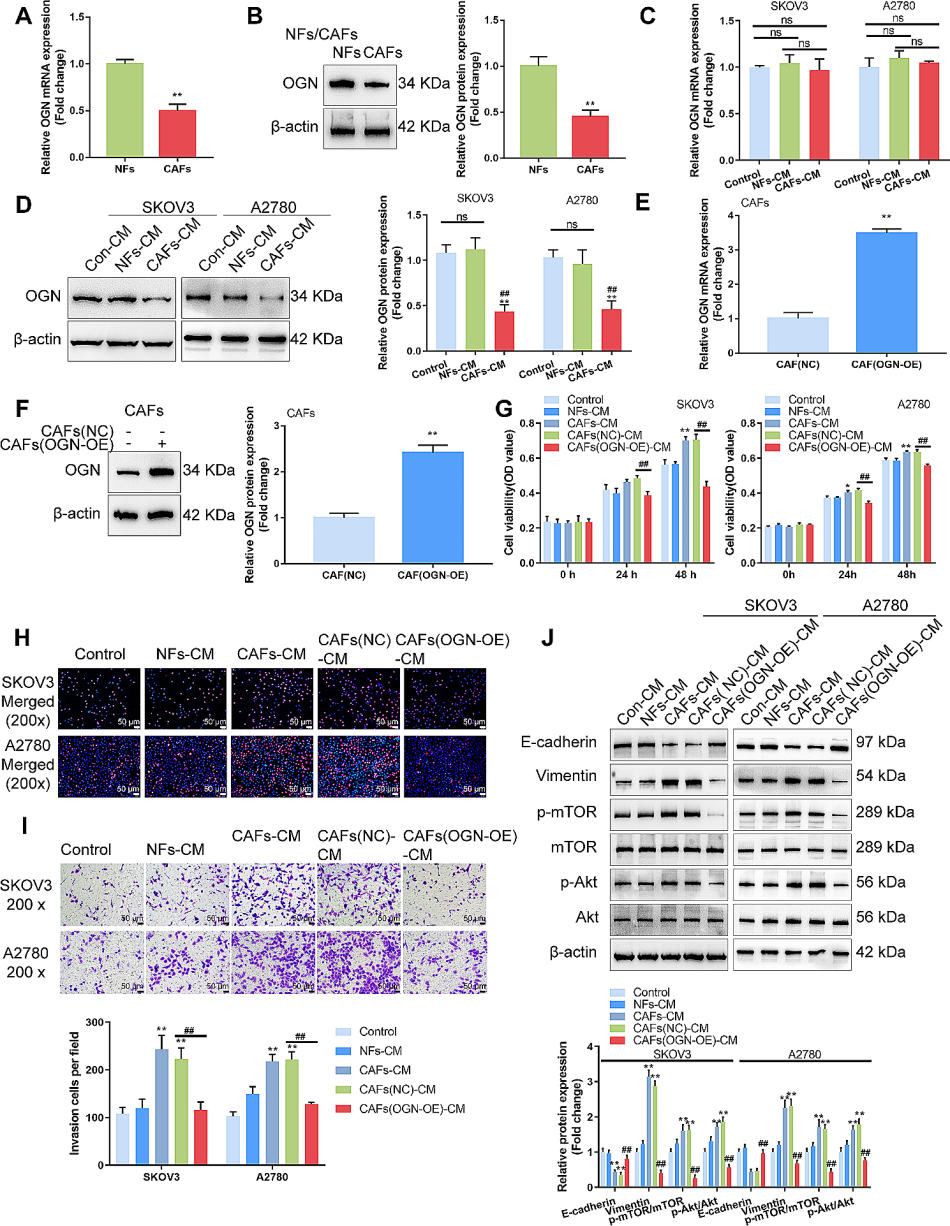
Culture with OGN-overexpressing CAFs-derived conditioned medium inhibits ovarian cancer cell aggressiveness (**A**–**B**) The mRNA expression and protein levels of OGN were examined in CAFs and NFs by qRT-PCR and Immunoblotting, respectively. (**C**–**D**) SKOV3 and A2780 cells were co-cultured with CAFs- or NFs-derived conditioned medium (CAFs-CM/NFs-CM) and examined for the mRNA expression and protein levels of OGN in SKOV3 and A2780 cells by qRT-PCR and Immunoblotting, respectively. (**E**–**F**) OGN overexpression was achieved in CAFs by transducing OGN overexpression vector (OGN-OE); OGN overexpression was confirmed by qRT-PCR and Immunoblotting, respectively. Then, SKOV3 and A2780 cells were cultured in control medium (con-CM), CAFs-CM, NF-CM, CAFs (lv-NC)-CM, or CAFs (OGN-OE)-CM, and examined for cell viability by MTT assay (**G**); DNA synthesis capacity by EdU (**H**); cell invasion by Transwell with chambers pre-coated with Matrigel (**I**); the protein levels of E-cadherin, vimentin, p-mTOR, mTOR, p-Akt, and Akt by Immunoblotting (**J**). **P < 0.01, compared with NFs or CAFs (NC) group; ##*P* < 0.01 compared with CAFs (NC)-CM group

Next, OGN mRNA and protein expression within isolated CAFs and NFs were determined; Fig. 2A-B showed that OGN mRNA and protein expression were dramatically downregulated within CAFs than those in NFs. Then, SKOV3 cells were co-cultured with CAFs- or NFs-derived conditioned medium (CAFs-CM/NFs-CM) and examined OGN mRNA and protein expression in SKOV3 and A2780 cells; as shown in Fig. 2C-D, OGN mRNA levels showed no significant changes among groups, whereas OGN protein levels were significantly downregulated in SKOV3 cells cultured in CAFs-CM. For investigating the specific effects of OGN, OGN overexpression was achieved in CAFs by transfecting OGN overexpression vector (OGN-OE); OGN overexpression was confirmed by qRT-PCR and Immunoblotting (Fig. 2E-F). Then, SKOV3 and A2780 cells were cultured in a control medium (con-CM), CAFs-CM, NFs-CM, CAFs (lv-NC)-CM, or CAFs (OGN-OE)-CM, and examined for cell phenotypes. Compared with the con-CM and NFs-CM group, CAFs-CM and CAFs (NC)-CM significantly promoted cell viability (Fig. 2G), DNA synthesis capacity (Fig. 2H), and cell invasion (Fig. 2I). Consistently, CAFs-CM and CAFs (NC)-CM inhibited E-cadherin protein contents, enhanced vimentin protein contents, and promoted the phosphorylation of mTOR and Akt (Fig. 2J). Conversely, CAFs (OGN-OE)-CM significantly attenuated the promotive effects of CAFs-CM and CAFs (lv-NC)-CM on cell viability, DNA synthesis capacity, and cell invasion. They also attenuated the changes in E-cadherin, vimentin, and the phosphorylation of mTOR and Akt caused by CAFs-CM or CAFs (lv-NC)-CM (Fig. 2G-J).

### miR-1290 directly targets OGN and inhibits OGN expression

**Fig. 3:**
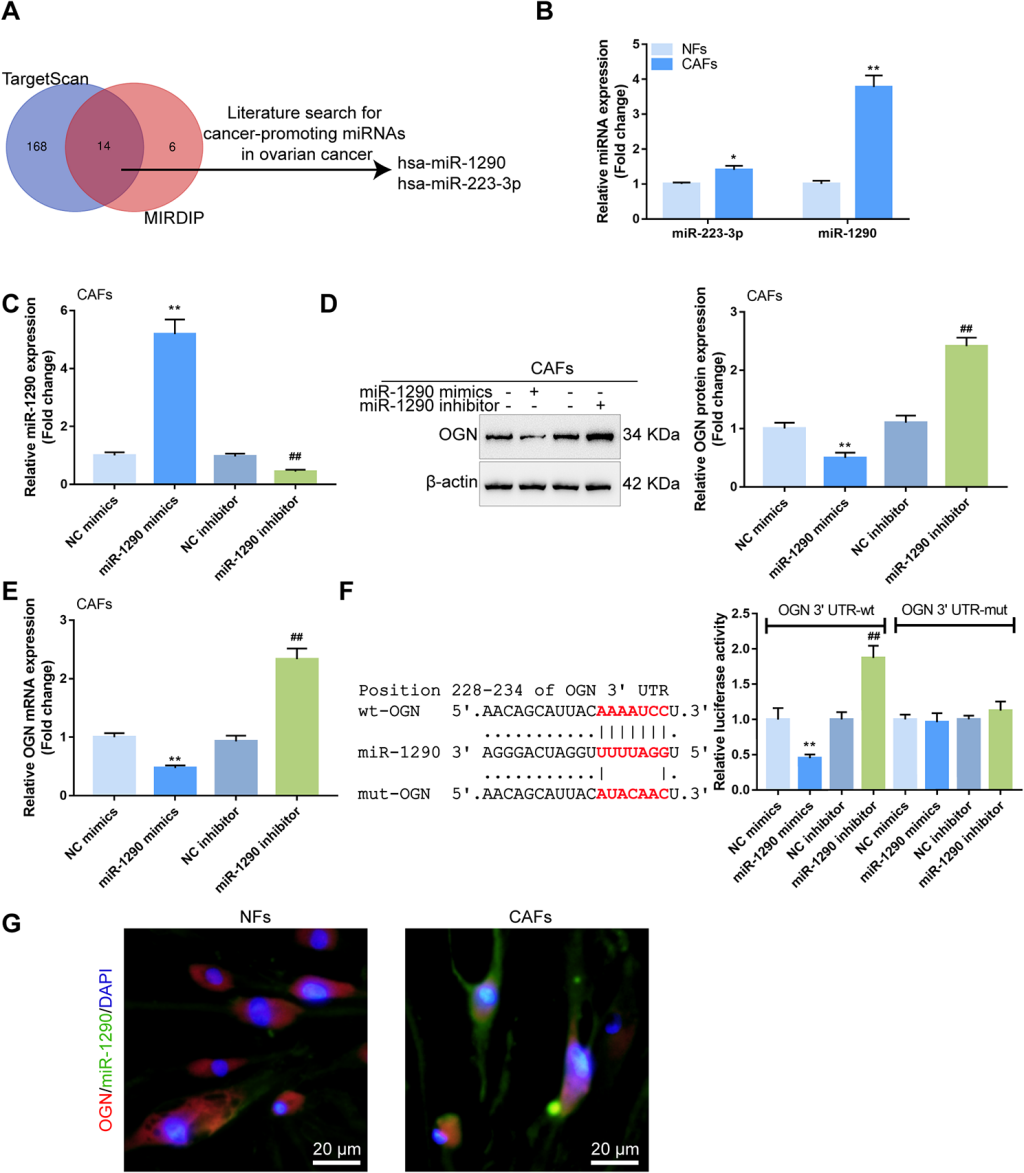
miR-1290 directly targets OGN and inhibits OGN expression (**A**) Targetscan, miRDIP, and literature search methods were used to search for miRNAs that might target OGN and serve as oncogenic miRNAs, and two miRNAs (miR-223-3p and miR-1290) were obtained. (**B**) The expression of miR-223-3p and miR-1290was examined in CAFs and NFs by qRT-PCR. (**C**–**D**) miR-1290 overexpression or inhibition was achieved in CAFs by transducing miR-1290 mimics or inhibitor; miR-1290 overexpression or inhibition was confirmed by qRT-PCR and Immunoblotting, respectively. (**E**) CAFs were transduced with miR-1290 mimics or inhibitor and examined for the mRNA expression by qRT-PCR. (**F**) Wild- and mutant-type OGN luciferase reporter vectors (wt-OGN/mut-OGN) were constructed as described in the M&M section. Then, wt-OGN or mut-OGN was co-transduced into 293T cells with miR-1290 mimics/inhibitor; the luciferase activity was determined. (**G**) The expression and location of miR-1290 and OGN in CAFs and NFs were determined by FISH assay. miR-1290 (green), OGN (red). Scale bar = 20 μm. *P < 0.05, **P < 0.01, compared with NC mimics; ##*P* < 0.01, compared with NC inhibitor

Since miRNAs target downstream mRNA and inhibit mRNA expression, next, Targetscan and miRDIP were used to search for miRNAs that might target OGN and inhibit OGN expression in CAFs; 14 miRNAs were predicted by both tools to target OGN (Fig. 3A). Among these 14 miRNAs, miR-1290 and miR-223-3p were previously reported to serve as oncogenic miRNAs [30–36]. In CAFs, miR-223-3p and miR-1290 expression showed to be remarkably increased, and miR-1290 showed to be dramatically upregulated (Fig. 3B). Next, miR-1290 promoter methylation level was detected in NFs and CAFs using MSP assay. Fig. S3 showed that the methylated level of the miR-1290 promoter was dramatically lower within CAFs than that in NFs. Hence, it was speculated that the high expression of miR-1290 in CAFs may be related to its promoter methylation. Moreover, the effect of miR-1290 on CAF functions in ovarian cancer remains unclear. Thus, miR-1290 was selected for subsequent experiments.

miR-1290 overexpression or inhibition was achieved in CAFs by transducing miR-1290 mimics or inhibitor; miR-1290 overexpression or inhibition was confirmed by qRT-PCR (Fig. 3C). In CAFs, miR-1290 overexpression downregulated, whereas miR-1290 inhibition upregulated OGN mRNA and protein expression (Fig. 3D-E). To confirm the binding of miR-1290 to OGN, wild- and mutant-type OGN luciferase reporter vectors (wt-OGN/mut-OGN) were constructed and co-transduced into 293T cells with miR-1290 mimics/inhibitors. When co-transduced with wt-OGN, miR-1290 overexpression was inhibited, whereas miR-1290 inhibition enhanced the luciferase activity; when co-transduced with mut-OGN, miR-1290 overexpression or miR-1290 inhibition failed to change the luciferase activity (Fig. 3F). Thus, miR-1290 might target OGN and inhibit OGN expression. Moreover, a FISH assay was performed to determine the expression and location of miR-1290 and OGN mRNA in CAFs and NFs. It was found that miR-1290 and OGN were mainly distributed in the cytoplasm of CAFs and NFs, and miR-1290 expression was present at higher levels in CAFs, while OGN was highly expressed in NFs (Fig. 3G).

### Effects of Mir-1290-overexpressing or -inhibited CAFs-derived conditioned medium on ovarian cancer cell aggressiveness

**Fig. 4:**
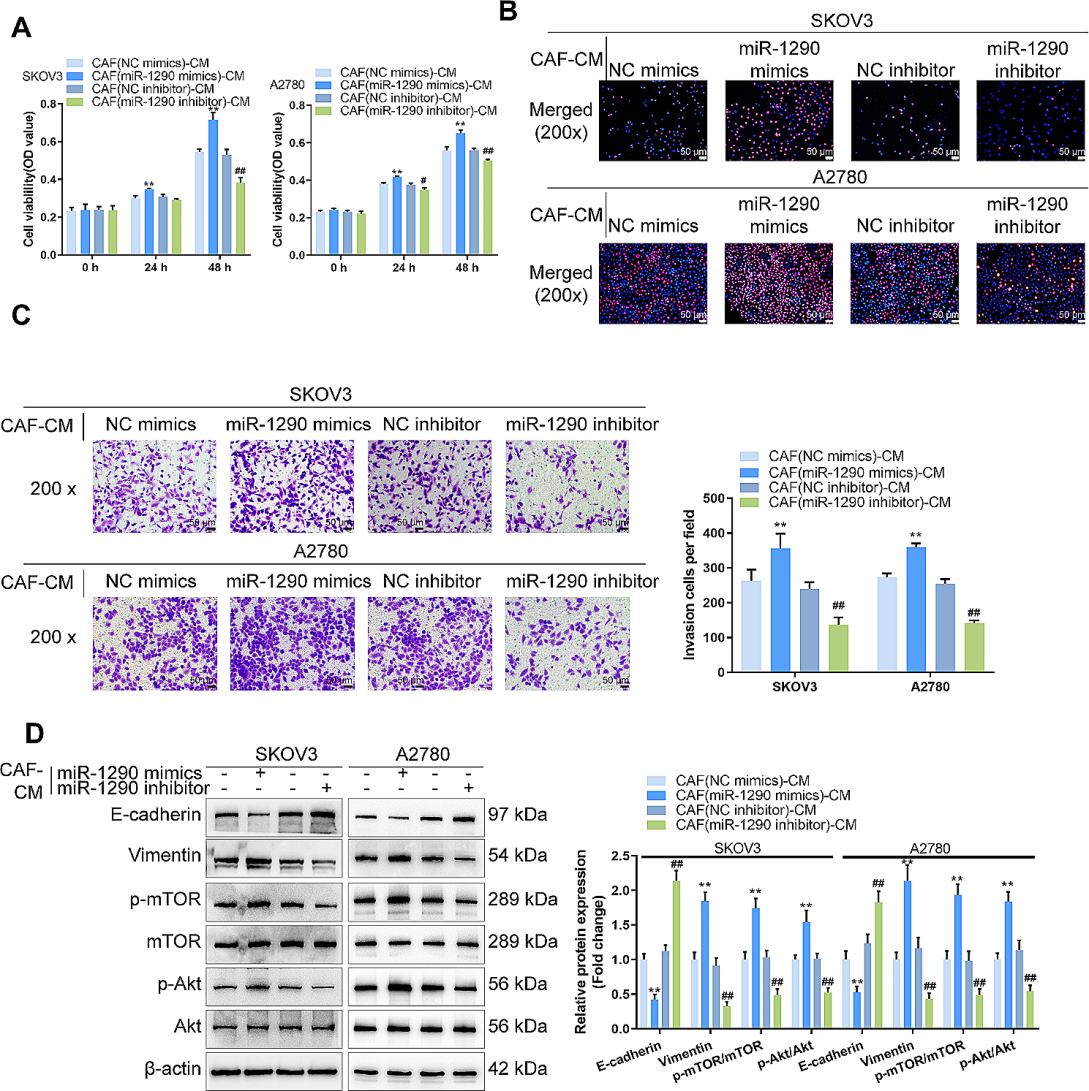
Effects of miR-1290-overexpressing or -inhibited CAFs-derived conditioned medium on ovarian cancer cell aggressiveness SKOV3 and A2780 cells were cultured in CAFs transduced with miR-1290 mimics or inhibitor and examined for cell viability by MTT (**A**); DNA synthesis capacity by EdU (**B**); cell invasion by Transwell with chambers pre-coated with Matrigel (**C**); the protein levels of E-cadherin, vimentin, p-mTOR, mTOR, p-Akt, and Akt by Immunoblotting (**D**). **P < 0.01, compared with CAFs (NC mimics)-CM group; ##*P* < 0.01 compared with CAFs (NC inhibitor)-CM group

Similarly, SKOV3 and A2780 cells in CAFs were subsequently cultured and transduced with miR-1290 mimics or inhibitor (CAF (miR-1290 mimics)-CM/CAF (miR-1290 inhibitor)-CM), and the cell phenotypes of SKOV3 and A2780 cells were examined. CAF (miR-1290 mimics)-CM significantly promoted, whereas CAF (miR-1290 inhibitor)-CM inhibited cell viability, DNA synthesis capacity, and cell invasion of SKOV3 and A2780 cells (Fig. 4A-C). Consistently, CAF (miR-1290 mimics)-CM decreased E-cadherin, increased vimentin, and promoted the phosphorylation of mTOR and Akt in SKOV3 and A2780 cells, whereas CAF (miR-1290 inhibitor)-CM exerted opposite effects (Fig. 4D).

### The miR-1290/OGN axis in CAFs modulates ovarian cancer cell aggressiveness

**Fig. 5:**
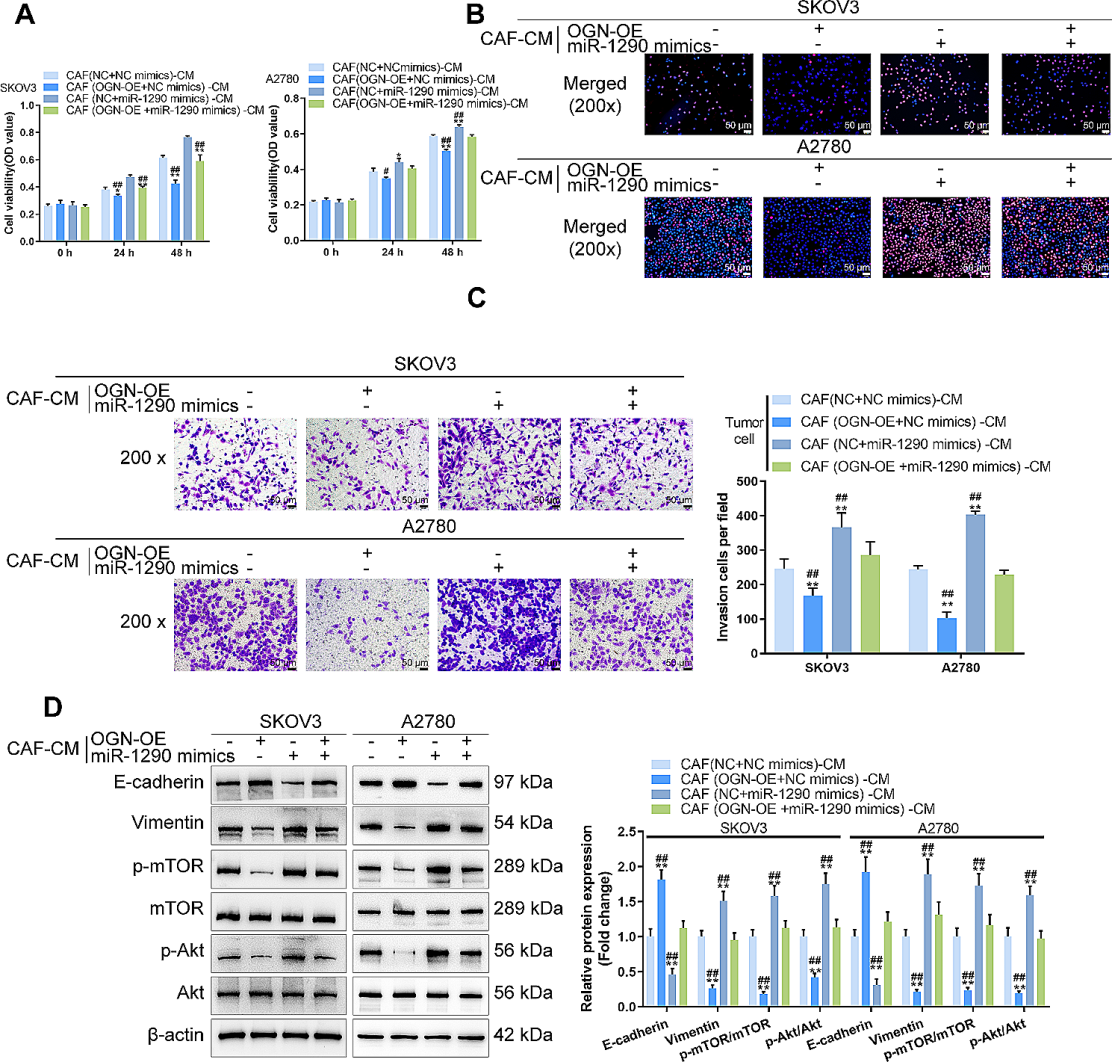
The miR-1290/OGN axis in CAFs modulates ovarian cancer cell aggressiveness SKOV3 and A2780 cells were cultured in CAFs co-transduced with miR-1290 mimics and OGN-OE and examined for cell viability by MTT (**A**); DNA synthesis capacity by EdU (**B**); cell invasion by Transwell with chambers pre-coated with Matrigel (**C**); the protein levels of E-cadherin, vimentin, p-mTOR, mTOR, p-Akt, and Akt by Immunoblotting (**D**). **P < 0.01, compared with CAFs (NC + NC mimics)-CM group; ##*P* < 0.01 compared with CAFs (OGN-OE + miR-1290 mimics)-CM group

Since miR-1290 targets OGN and inhibits OGN expression, next, SKOV3 and A2780 cells were cultured in CAFs co-transduced with miR-1290 mimics, and OGN-OE and cell phenotypes were investigated. OGN overexpression in CAFs was inhibited, whereas miR-1290 overexpression promoted cell viability, DNA synthesis capacity, and cell invasion of SKOV3 and A2780 cells (Fig. 5A-C); the effects of miR-1290 overexpression were partially reversed by OGN overexpression (Fig. 5A-C). OGN overexpression in CAFs increased E-cadherin, decreased vimentin, and inhibited the phosphorylation of mTOR and Akt in SKOV3 and A2780 cells, whereas miR-1290 overexpression in CAFs exerted opposite effects; the effects of miR-1290 overexpression were partially reversed by OGN overexpression (Fig. 5D).

### In vivo effects of the miR-1290/OGN axis in CAFs modulates tumor growth in nude mice models

**Fig. 6:**
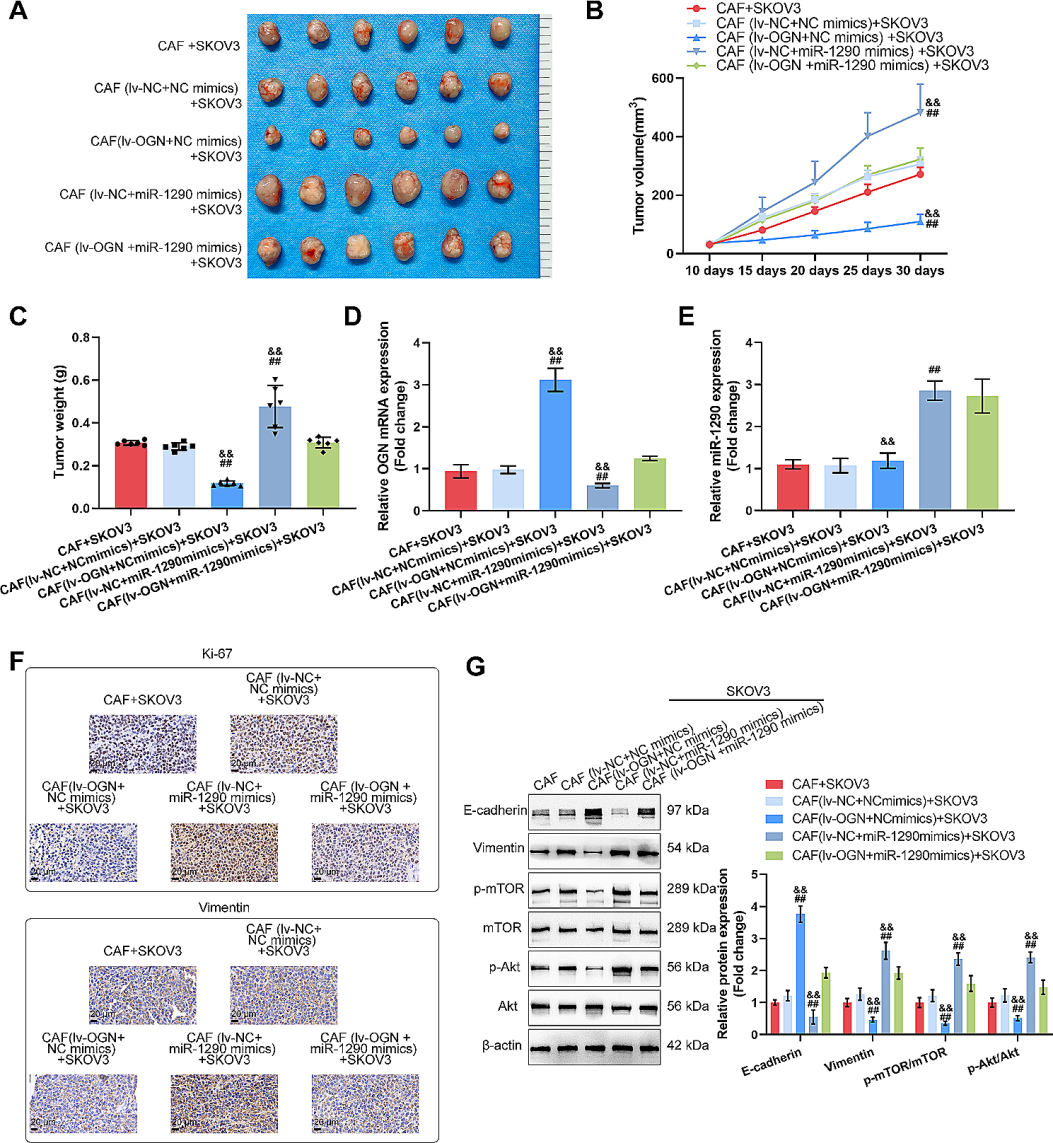
The miR-1290/OGN axis in CAFs modulates tumorigenic capacity of ovarian cancer cell in vivo Xenograft transplanted tumor models were established in nude mice by injecting a mixture of SKOV3 cells with CAFs or infected CAFs (infected with miR-1290 mimics or lv-OGN) and nude mice were separated into five groups (*n* = 6 per group): the SKOV3 cells + CAFs group, the SKOV3 cells + CAFs (lv-NC + NC mimics) group, the SKOV3 cells + CAFs (lv-OGN + NC mimics) group, the SKOV3 cells + CAFs (lv-NC + miR-1290 mimics) group, the SKOV3 cells + CAFs (lv-OGN + miR-1290 mimics) group. (**A**) Images of the tumors in each group. (**B**) Tumor volumes were measured every five days from the 10th day of the experiment. (**C**) Tumor weight were determined at the 30th day. (**D**–**E**) The mRNA levels of OGN and miR-1290 in mice tumor tissues were detected by qRT-PCR. (**F**) Ki67 and Vimentin levels in tumor tissues were examined using IHC staining. Scale bar = 20 μm. (**G**) The protein levels of E-cadherin, vimentin, p-mTOR, mTOR, p-Akt, and Akt in tumor tissues were determined using immunoblotting. *P < 0.05, **P < 0.01, compared between SKOV3 cells + CAFs group and SKOV3 cells + CAFs (lv-NC + NC mimics); ##*P* < 0.01 compared with SKOV3 cells + CAFs (lv-NC + NC mimics) group; &&*P* < 0.01 compared with SKOV3 cells + CAFs (lv-OGN + miR-1290 mimics) group

To investigate the role of the miR-1290/OGN axis in mediating CAFs functions on ovarian cancer cells in vivo, xenograft models were established in nude mice by subcutaneously injecting the mixture of SKOV3 cells along with CAFs or transfected CAFs (miR-1290 mimics/lv-OGN). Figure 6A-C demonstrated that the tumor volumes and weights of tumors derived from the mixture of cancer cells and CAFs-miR-1290 mimics were significantly greater than those derived from the mixture of cancer cells and CAFs-NC, whereas those derived from the mixture of cancer cells and CAFs-lv-OGN were reduced than those derived from CAFs-NC; the effects of miR-1290 overexpression in CAFs were partially reversed by OGN overexpression (Fig. 6A-C). miR-1290 overexpression in CAFs increased miR-1290 expression and inhibited OGN expression in tumor tissues and OGN overexpression in CAFs increased OGN expression (Fig. 6D-E). IHC results indicated that the protein levels of Ki-67 and vimentin were elevated in CAFs-miR-1290 mimics tumor tissues compared with CAFs-NC tumor tissues; while in CAFs-lv-OGN tumor tissues, Ki-67 and vimentin showed to be decreased compared with CAFs-NC tumor tissues (Fig. 6F). Finally, miR-1290 overexpression decreased E-cadherin, increased vimentin, and the phosphorylation of mTOR and Akt in CAFs-miR-1290 mimics tumor tissues, while OGN overexpression exerted opposite effects in CAFs-lv-OGN tumor tissues; the effects of miR-1290 overexpression were partially reversed by OGN overexpression (Fig. 6G).

## Discussion

This study isolated CAFs and NFs from tumor-containing and normal omentum, respectively. The downregulation of OGN in CAFs was observed. OGN overexpression in CAFs significantly inhibited ovarian cancer cell viability, DNA synthesis, and cell invasion. It also changed EMT markers and promoted mTOR and Akt phosphorylation in ovarian cancer cells. miR-1290 targeted OGN and inhibited OGN expression. miR-1290 overexpression in CAFs significantly promoted ovarian cancer cell viability, DNA synthesis, and cell invasion; besides, miR-1290 overexpression in CAFs also changed EMT markers and promoted mTOR and Akt phosphorylation within ovarian carcinoma cells. Then, when ovarian cancer cells were cultured in a conditioned medium derived from CAFs co-transduced with miR-1290 mimics and OGN-OE, the effects of miR-1290 overexpression were partially reversed by OGN overexpression. Finally, in nude mouse xenograft tumor models, OGN overexpression in CAFs suppressed tumor growth, whereas miR-1290 overexpression in CAFs increased tumor growth.

CAFs differ from their normal counterparts by the differential expression of markers such as α-smooth muscle actin (α-SMA), fibroblast activation protein (FAP), growth factors TGFβ1, TGFβ2, PDGF, platelet-derived growth factor receptor PDGFRα, PDGFRβ), βFGF, and periostin, neovascularization marker VEGF, chemokine/cytokines IL6 and CXCL12, ECM proteins including tenascin-C, and neuron glial antigen-2, and structural proteins vimentin, desmin, and fibroblast specific protein-1 [37–41]. In this study, CAFs isolated from tumor-containing omentum showed different α-SMA levels compared with NFs isolated from normal omentum. Commonly, CAFs undergo an activation process associated with the secretion of growth factors, cytokines, and CAF-specific proteins and induce the production of ECM proteins, thereby promoting the growth of tumor cells and the formation of new blood vessels and conferring the resistance to chemotherapy [4–7, 42]. Thus, elevated α-SMA levels suggest that ovarian cancer-derived CAFs might play a role in ovarian cancer carcinogenesis.

As one of the critical and abundant components in the tumor microenvironment, CAFs have been involved in the malignant behaviors of tumor cells [43, 44]. Herein, online datasets and experimental investigation both indicated the downregulation of OGN in CAFs. As mentioned earlier, OGN serves as an anti-tumor factor inhibiting the capacity of tumor cells to proliferate, invade, and/or migrate in other cancers. Within breast carcinoma, OGN suppresses the capacity of tumor cells to proliferate and invade through the PI3K/Akt/mTOR signaling [16]. In colorectal cancer, OGN reversed EMT and invasive abilities through the EGFR/Akt pathway [17]. Herein, conditioned culture medium derived from OGN-overexpressed CAFs significantly inhibited ovarian cancer cell viability, DNA synthesis, and cell invasion. Moreover, epithelial marker E-cadherin was upregulated, mesenchymal marker vimentin was downregulated, and the phosphorylation of mTOR and Akt was inhibited. Thus, OGN overexpression in CAFs could inhibit the malignant behaviors of ovarian cancer cells, possibly by affecting cancer cell proliferation and adhesion.

As mentioned earlier, OGN expression is downregulated in CAFs compared with NFs. MiRNAs attracted our attention because of their inhibitory effects on downstream mRNAs through targeting [20, 45], miRNAs attracted our attention. Consistent with the online tool prediction, experimental investigation also indicated that miR-1290 could target OGN and inhibit OGN expression. As mentioned earlier, miR-1290 serves as an oncogenic miRNA, promoting the capacity of tumor cells to proliferate, invade, and migrate within colorectal cancer [30], esophageal squamous cell carcinoma [32], and non-small cell lung cancer [33]. Although these studies reported the oncogenic effects of miR-1290 directly on tumor cells, the indirect effects of miR-1290 upon tumor cells through CAFs are still unclear. Herein, miR-1290 overexpression in CAF aggravated the malignant behaviors of ovarian cancer cells, whereas miR-1290 inhibition exerted opposite effects. Furthermore, OGN overexpression in CAF significantly attenuated the roles of miR-1290 overexpression in CAF. Moreover, the crosstalk between CAFs and cancer cells are often mediated by extra-cellular signals including extracellular vesicles (EVs, including exosomes) [46]. EVs are a group of heterogeneous nanometer-sized vesicles that are released by all types of cells and serve as functional mediators of cell-to-cell communication [47]. Additionally, EVs or exosomes can act as mediators for intercellular crosstalk by the delivery of biomolecules, such as proteins, microRNAs (miRNAs), mRNA, DNA) and lipids [48, 49]. Hence, we speculate that CAFs may secrete extracellular vesicles or exosomes containing miR-1290 and OGN, thereby affecting the expression of E-cadherin, mTOR and AKT in ovarian cancer cells and mediating the proliferation and metastasis of ovarian cancer cells. Previous studies also reached similar conclusions. Wang et al. [50] demonstrated that cancer associated fibroblasts secreted exosomal miR-1290 contributes to prostate cancer cell growth and metastasis via targeting GSK3β. Bai et al. [51] suggested that COX-2/exo-miR-1290/CUL3 is suggested as a novel signaling pathway for mediating CAFs activation and tumor progression in lung adenocarcinoma. Taken together, miR-1290 might be the reason for OGN downregulation in CAFs, and affect the crosstalk between CAFs and cancer cells, thereby modulating the malignant behaviors of ovarian cancer cells. The specific mechanism needs further verification in follow-up experiments.

In conclusion, a miRNA/mRNA axis in ovarian cancer CAFs modulating the proliferative and invasive abilities of ovarian cancer cells was demonstrated, possibly through the Akt/mTOR pathway.

### Electronic supplementary material

Below is the link to the electronic supplementary material.

Supplementary Material 1

Supplementary Material 2

Supplementary Material 3

Supplementary Material 4

## Data Availability

This study analyzed online datasets to confirm OGN expression in ovarian cancer based on GSE40595 and GSE38666.
