# Supplementary figures and images for: The miR-1290/OGN axis in ovarian cancer-associated fibroblasts modulates cancer cell proliferation and invasion
Source: J Ovarian Res. 2024 Feb 24;17:52. doi: 10.1186/s13048-024-01364-w (PMC10893657; doi:10.1186/s13048-024-01364-w)

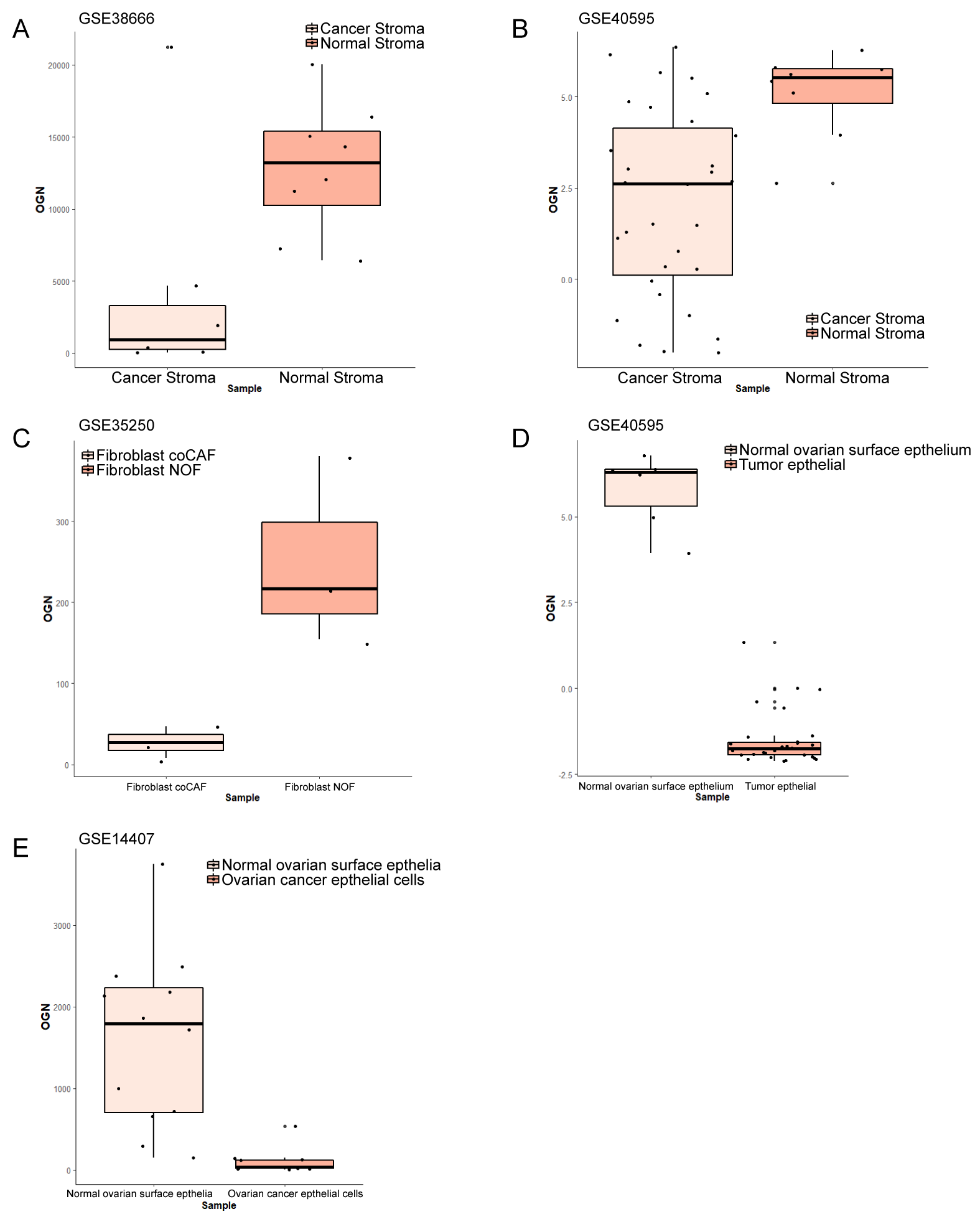

Supplement: Supplementary file 1 — Supplementary Material 1 [file 13048_2024_1364_MOESM1_ESM.png]

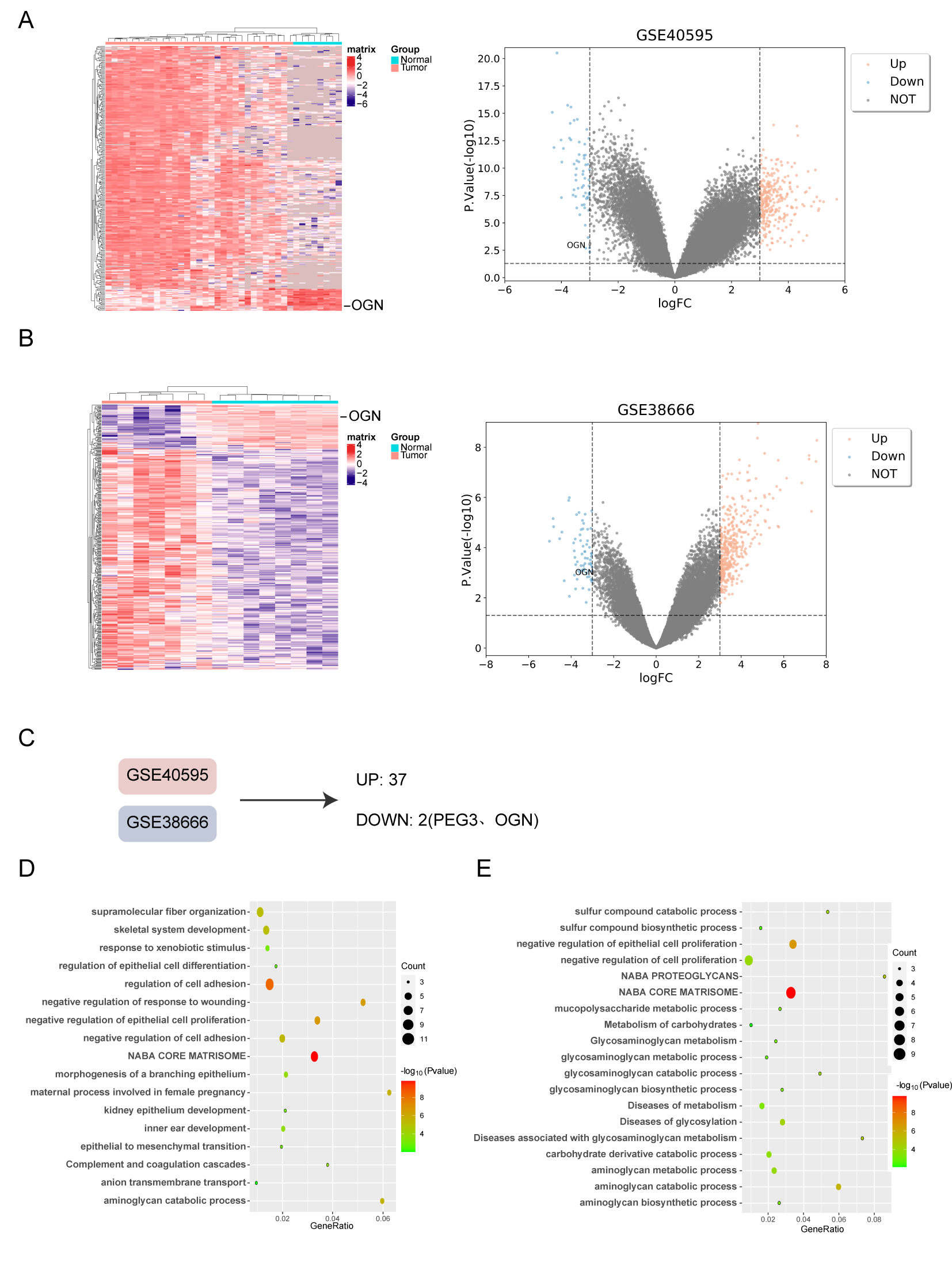

Supplement: Supplementary file 2 — Supplementary Material 2 [file 13048_2024_1364_MOESM2_ESM.png]

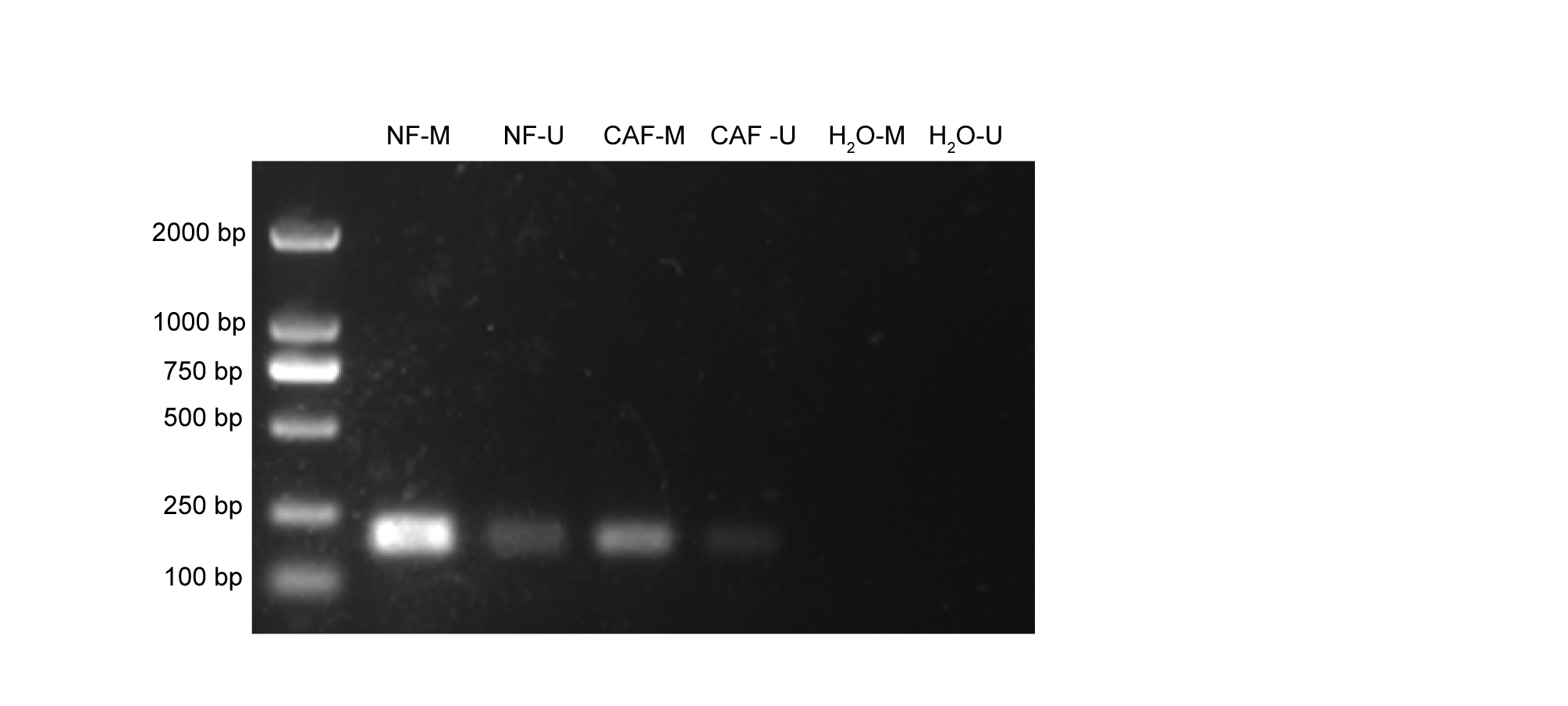

Supplement: Supplementary file 3 — Supplementary Material 3 [file 13048_2024_1364_MOESM3_ESM.png]
