# Supplementary material for: The miR-1290/OGN axis in ovarian cancer-associated fibroblasts modulates cancer cell proliferation and invasion
Source: J Ovarian Res. 2024 Feb 24;17:52. doi: 10.1186/s13048-024-01364-w (PMC10893657; doi:10.1186/s13048-024-01364-w)
Supplement: Supplementary file 4 — Supplementary Material 4 [file 13048_2024_1364_MOESM4_ESM.docx]

**Table S1. the primer sequence for the study**

| **Category** | **Froward (5'-3')** | **Reverse (5'-3')** |
| --- | --- | --- |
| RT-PCR  OGN | TAAAGCCAGCACCACCAAC | TTCCATCCAGGTATTTATCC |
| RT-PCR  miR-223-3p | RT：GTCGTATCCAGTGCGTGTCGTGGAGTCGGCAATTGCACTGGATACGACTGGGGT  F：GCCGTGTCAGTTTGTCAAAT | CAGTGCGTGTCGTGGA |
| RT-PCR  miR-1290 | RT：  GTCGTATCCAGTGCGTGTCGTGGAGTCGGCAATTGCACTGGATACGACTCCCTG  F：GCCGTGGATTTTTGGAT | CAGTGCGTGTCGTGGA |
| RT-PCR  GAPDH | ACAGCCTCAAGATCATCAGC | GGTCATGAGTCCTTCCACGAT |
| RT-PCR  U6 | CTCGCTTCGGCAGCACA | AACGCTTCACGAATTTGCGT |
| NC | / | / |
| OGN-OE | ctagcgtttaaacttaagcttATGAAGACTCTGCAGTCTACACTTCTC | tgctggatatctgcagaattcTTAAAAGTATGACCCTATCGGTAATCTT |
| mimics NC | UUCUCCGAACGUGUCACGUTT | ACGUGACACGUUCGGAGAATT |
| miR-1290 mimics | UGGAUUUUUGGAUCAGGGA | CCUGAUCCAAAAAUCCAUU |
| inhibitor NC | CAGUACUUUUGUGUAGUACAA |  |
| miR-1290 inhibitor | UCCCUGAUCCAAAAAUCCA |  |
| lv-NC | / | / |
| lv-OGN-OE | ctaccggactcagatctcgagATGAAGACTCTGCAGTCTACACTTCTC | gtaccgtcgactgcagaattcTTAAAAGTATGACCCTATCGGTAATCTT |
| Wt-OGN 3’UTR | aattctaggcgatcgctcgagAGTATTGGTTTAATATTAACCTTGTATCTCAT | attttattgcggccagcggccgcTAAAGGCCAGGAAAGGCAAA |
| Mut-OGN 3’UTR | GCATTACttttaggTAGTTTATACTAGACTACCATTTAAAAATCATGT | CTAcctaaaaGTAATGCTGTTTAAATATTTTCATATTACTTTG |
| Methylation Specifc PCR  miR-1290 promoter  Methylated primer | GCCATTGAGGCAGTGCTTGGCAGTGG | CCCAGAGAGGGTGAGTAACCTGCCC |
| Methylation Specifc PCR  miR-1290 promoter  unmethylated primer | TGGTAGTGGTATTTTTGGGAGTAGT | TCCATATAAAAACATAAAAAAACCCA |
